# Impact of COVID-19 on routine immunisation in South-East Asia and Western Pacific: Disruptions and solutions

**DOI:** 10.1016/j.lanwpc.2021.100140

**Published:** 2021-04-21

**Authors:** Rebecca C. Harris, Yutao Chen, Pierre Côte, Antoine Ardillon, Maria Carmen Nievera, Anna Ong-Lim, Somasundaram Aiyamperumal, Chan Poh Chong, Kiruthika Velan Kandasamy, Kuharaj Mahenthiran, Ta-Wen Yu, Changshu Huang, Clotilde El Guerche-Séblain, Juan C. Vargas-Zambrano, Ayman Chit, Gopinath Nageshwaran

**Affiliations:** a Sanofi Pasteur, 38 Beach Road, #18-11 South Beach Tower, Singapore 189767, Singapore; b London School of Hygiene and Tropical Medicine, Keppel Street, London WC1E 7HT, UK; c Sanofi Pasteur China, 7F HP Plaza, 112 Jianguo Rd, ChaoYang District, Beijing 100022, China; d Sanofi Pasteur, 14 Espace Henry Vallée, 69007 Lyon, France; e Current affiliation: Institute of Pharmaceutical and Biological Sciences, Claude Bernard Lyon University, 8 avenue Rockefeller, 69373 Lyon, France; f Division of Infectious and Tropical Disease, Department of Pediatrics, University of the Philippines – Philippine General Hospital, Manila 1000, Philippines; g Department of Developmental and Behavioural Pediatrics, D'Soul Child Development Centre and Kanchi Kamakoti Childs Trust Hospital, Chennai 600034, India; h Division of General Ambulatory Paediatrics and Adolescent Medicine, Department of Paediatrics, National University Children's Medical Institute, National University Hospital, 1E Kent Ridge Road, NUHS Tower Block, Singapore 119228, Singapore; i Sanofi Pasteur, Rain Tree Place, Mc Nichols Road, Chetpet, Chennai 600031, India; j Sanofi Pasteur, New World International Trade, No.568, Jianshe Avenue, Jianghan District, Wuhan City 430000, China; k Sanofi Pasteur, Discovery Drive Swiftwater, PA 18370, USA; l Leslie Dan Faculty of Pharmacy, University of Toronto, Toronto, ON M5S 3M2, Canada

**Keywords:** Routine vaccination, Immunisation, Disruption, COVID-19, Vaccines, Asia, Vaccination coverage rates, Public health, Preventive care

## Abstract

**Background:**

Data on COVID-19-induced disruption to routine vaccinations in the South-East Asia and Western Pacific regions (SEAR/WPR) have been sparse. This study aimed to quantify the impact of COVID-19 on routine vaccinations by country, antigen, and sector (public or private), up to 1 June 2020, and to identify the reasons for disruption and possible solutions.

**Methods:**

Sanofi Pasteur teams from 19 countries in SEAR/WPR completed a structured questionnaire reporting on COVID-19 disruptions for 13–19 routinely delivered antigens per country, based on sales data, government reports, and regular physician interactions. Data were analysed descriptively, disruption causes ranked, and solutions evaluated using a modified public health best practices framework.

**Findings:**

95% (18/19) of countries reported vaccination disruption. When stratified by country, a median of 91% (interquartile range 77–94) of antigens were impacted. Infancy and school-entry age vaccinations were most impacted. Both public and private sector healthcare providers experienced disruptions. Vaccination rates had not recovered for 39% of impacted antigens by 1 June 2020. Fear of infection, movement/travel restrictions, and limited healthcare access were the highest-ranked reasons for disruption. Highest-scoring solutions were separating vaccination groups from unwell patients, non-traditional vaccination venues, virtual engagement, and social media campaigns. Many of these solutions were under-utilised.

**Interpretation:**

COVID-19-induced disruption of routine vaccination was more widespread than previously reported. Adaptable solutions were identified which could be implemented in SEAR/WPR and elsewhere. Governments and private providers need to act urgently to improve coverage rates and plan for future waves of the pandemic, to avoid a resurgence of vaccine-preventable diseases.

**Funding:**

Sanofi Pasteur.

**Research in Context**

*Evidence before this study*

Prior to conducting our study, we monitored journals in the fields of vaccines, infectious diseases, and childhood diseases. We also sought information from publications by and the websites of relevant bodies and networks including the World Health Organization, the Vaccine Alliance (Gavi), the US Centers for Disease Control, and the IMmunising PRegnant women and Infants NeTwork (IMPRINT). One systematic review has been published on this topic, but for Asia and the Pacific the review only reported publications from Pakistan. Data on COVID-19-induced disruption to routine vaccinations were sparse, particularly in terms of coverage for the South-East Asia and Western Pacific regions (SEAR/WPR), and did not explore impact by age, sub-national disruption, or sector.

*Added value of this study*

This study provides the first assessment of the impact of the COVID-19 pandemic on routine vaccinations in SEAR/WPR by country, antigen, and healthcare sector. The results showed that COVID-19-induced disruption of routine vaccination was more widespread than previously reported. Vaccinations of infants and school-entry age children were most disrupted; within-country differences were evident; and both public and private sector delivery was disrupted. The study also identifies and ranks the reasons for disruptions in order to understand the situation better, and provides a list of solutions designed and implemented by countries to overcome these barriers to immunisation. Overarching themes assessed using a public health best practices framework uncovered several high-scoring solutions that were reported to be used in only a few countries in the region.

*Implications of all the available evidence*

The study has provided valuable data on the extent of disruption by country, antigen, age and sector, thus substantively contributing to an important evidence gap in the literature concerning COVID-19-induced disruptions to vaccination in SEAR/WPR. The results show that urgent action is needed both by governments and by private healthcare providers to improve vaccination coverage rates in order to prevent future increases in vaccine-preventable diseases. To aid such action, reasons for the disruptions were identified, and the solutions presented could be implemented in SEAR/WPR and beyond to minimise disruptions during future waves of the pandemic. The demonstration that both public and private sector providers were impacted highlights the need for private sector providers to be included in efforts to minimise disruption. The limited availability and quality of data on vaccine coverage rates points to the need for more robust and real-time reporting of this parameter across the different immunisation cohorts. The evidence on variations in impact by age, national income category, and within countries provides pointers for targeted action.

Alt-text: Unlabelled box

## 1 Introduction

The COVID-19 pandemic has been unprecedented in its scale and impact and has necessitated measures such as regional or country-wide lockdowns, travel restrictions, and social distancing [1,2]. Even where these restrictions have successfully reduced transmission, further waves of COVID-19 may require their reimposition. However, these measures have led to unintended health consequences, including disruption of routine immunisation services [3,4].

Timely vaccination is widely accepted as a highly successful public health intervention. In the South-East Asia region (SEAR), for example, immunisation has eliminated transmission of wild polio and maternal and neonatal tetanus, and has dramatically reduced the prevalence of measles, Japanese encephalitis, and hepatitis B (HepB) [5]. Disruption to access to vaccines has the potential for serious public health impact [6]. Given that routine immunisation against infectious diseases is a core health service, there is a clear need to understand disruptions due to COVID-19 and to find the right balance between vaccinating children with life-saving vaccines, while also protecting against COVID-19 [7]. Some information on disruptions and the reasons for disruptions is available at the global level. The World Health Organization (WHO) and Gavi (the Vaccine Alliance) reported on disruption in Gavi-eligible countries as of 16 June 2020 [3], and the WHO has carried out three pulse surveys (i.e. rapid surveys not replacing regular scheduled surveillance) [8], [9], [10]. The first reported that 64% of 107 countries globally reported vaccination disruption at 29 April 2020 [8]. However, few SEAR/Western Pacific Region (WPR) countries participated in these surveys, which focused mostly on public sector reporting. The second WHO survey reported that, as of 5 June 2020, 55% of 11 surveyed countries in SEAR/WPR reported disruption to facility-based vaccination services and 45% to outreach vaccination campaigns [9]. The IMmunising PRegnant women and Infants NeTwork (IMPRINT) surveyed healthcare professionals in 18 countries in April 2020, but included only two countries from SEAR/WPR (India and Nepal) [11].

Published articles describe the suspension of routine vaccination services at the global level [12], and a resultant resurgence in preventable diseases [13,14]. However, there are very few published data exploring the effects of COVID-19 on vaccination delivery specifically in SEAR/WPR, aside from brief mentions of individual countries. In a systematic review of the impact of COVID-19 on immunisation programmes globally [15], the only Asian or Pacific country with publications identified was Pakistan, which experienced reductions in routine vaccinations in 2020, particularly during lockdown periods [16,17]. For example, 40 million children in Pakistan missed their polio vaccination between April and June 2020, when all mass vaccination programs were suspended in the country [14], and GAVI data indicated a reduction in March/April for diphtheria-tetanus-pertussis vaccine (DTP) coverage of 49%, 26% for oral polio vaccine (OPV), 23% for measles and 22% for bacille Calmette-Guérin (BCG) [18]. A study in the Sindh region found a 52.5% decline in the average daily number of childhood vaccinations during lockdown, of which BCG was the most impacted, and rural areas, urban slums and polio-endemic areas were most affected [19]. Similarly, in Karachi a 53% drop in daily immunisations for 0-23 month-olds was observed during lockdown, and disruptions continued beyond lockdown with 27% reduction still in the two months after lockdown [17]. The authors of the systematic review noted that there are very few studies on the impact of COVID-19 on immunisation coverage, or on barriers to immunisation from the users’ or providers’ perspective [15]. Thus, we need a better understanding of disruptions to routine immunisation in Asia and the Pacific – including that provided by both the public and private sectors, given that private providers play an important role in healthcare delivery in this region [20].

Quantification of the problem is the first step, but most important for public health is understanding the root causes of disruptions and identifying potential solutions. Solutions published to date have focused primarily on health service management of the pandemic itself, for example [21], with few published solutions aimed specifically at minimising disruption of routine vaccination [11]. The current study was designed and conducted by Sanofi Pasteur's Epidemiology and Medical Affairs teams, with a primary objective to quantify the impact of COVID-19 on routine vaccinations by country and by antigen in SEAR/WPR, and to identify the causes of disruptions and possible solutions. The overall aim was to help countries minimise COVID-related disruptions to routine immunisation during future waves of the pandemic.

## 2 Methods

### 2.1 Study design

A cross-sectional structured questionnaire was completed by Sanofi Pasteur country teams in the SEAR/WPR region between 24 June and 10 July 2020 (Supplementary Table 1), covering the period from the start of the pandemic (generally February/March 2020) up to 1 June 2020. Questionnaires were sent to the country medical head, and were completed with support from colleagues from medical affairs, epidemiology, market access, sales, public affairs, and any other relevant functions as needed.

Geographical entities with their own independent public health decision-making bodies participated separately and are referred to as ‘countries’ for the purpose of this study. The 19 participating countries (Supplementary Figure 1) were classified by World Bank (WB) income status [22], as follows: eight high-income (HI) (Australia, Brunei, Hong Kong, Japan, New Zealand, South Korea, Singapore, Taiwan); four upper-middle-income (UMI) (China, Indonesia, Malaysia, Thailand); and seven lower-middle-income (LMI) (Cambodia, India, Myanmar, Nepal, Pakistan, Philippines, Vietnam) countries.

The 19 countries in our study represented 19/41 (46%) of countries in the covered regions. The denominator consists of 38 SEAR/WPR countries as listed by WHO [23], plus Hong Kong and Taiwan (counted in our study as countries) and Pakistan (included in our study). Importantly, our study covered a population of 3.9 billion, representing 94% of the total population of 4.2 billion in SEAR/WPR plus Pakistan [24].

As the study did not gather patient or individual level data or involve any interventions, formal ethical approval was not sought. Data were mostly open source or company data, and informed consent was sought from those filling in the survey.

### 2.2 Survey description

The questionnaire was piloted by the medical teams in India and Singapore to streamline questions and ensure consistency in interpretation. The final questionnaire comprised four sections: 1) routine immunisation impacted by COVID-19; 2) vaccination coverage rates (VCRs); 3) reasons for disruption; and 4) measures to reduce disruption. Responses were based upon sales data, government reports and public health institution websites, press releases, and regular physician interactions. Routine vaccinations were defined as those recommended by WHO, as adopted in the given countries. Seventeen antigens were listed (Supplementary Table 1) and respondents could include additional country-specific, routinely delivered antigens. For combination vaccines, respondents replied for each antigen contained in the vaccine. ‘Impacted’ was defined as a change in the number of people receiving a given vaccine during COVID-19, and responses were stratified by antigen and age group. For each antigen respondents could choose between ‘impacted’, ‘not impacted’, ‘information not available’, and ‘not applicable’. ‘Disruption’ was defined as a reduction of any size in the number of people receiving the vaccine.

Age groups were adapted from the US Centers for Disease Control and Prevention's categorisation [25]. These were: early infancy (0–8 weeks), infants and toddlers (9 weeks to 23 months; subsequently referred to as ‘infancy’), preschool and school-entry age (2–6 years; subsequently referred to as ‘school-entry age’), children and adolescents (7–17 years), and adults and elderly (≥18 years). For each antigen, respondents replied separately by age group, so that estimates could be based on age-specific responses.

Respondents were asked to report any local geographical variations in impact on antigens and in the effect on public and private vaccination delivery, the VCR before COVID-19 disruptions, the lowest VCR during COVID-19 up to 1 June, and whether VCR had returned to pre-COVID-19 levels as of 1 June 2020. VCR data were classified as ‘reported’, based on publicly available data, or ‘estimated’, based on sales data, press releases and/or interactions with healthcare providers (HCPs). VCR data were reported as point-in-time estimates and were not annualised. Respondents were also asked to identify and rank reasons for disruption (listed in Supplementary Table 1 and Fig. 4), and to share measures undertaken to minimise vaccination disruption by three main sectors (public, private healthcare providers, and vaccine companies).

### 2.3 Data analysis

Descriptive statistical methods were used to analyse the impact on immunisation, with median and interquartile ranges estimated for the proportions and numbers of antigens or countries impacted, including stratification by age and WB income stratum. The denominator for calculating percentages was the number of antigens for which countries provided any answer except ‘not applicable’. Significance tests were conducted for differences between age groups and WB country income levels using Kruskall–Wallis (K–W), with post-hoc comparisons between groups performed by the Dunn test with Bonferroni correction. Where data were available, the proportion of antigens impacted in the public and private sectors were estimated.

Reasons for disruption were ranked by weighted average ranking, in which the top-ranked reason was assigned a weight of 9, the second-ranked 8, etc., and ‘not applicable’ 0.

Solutions to reduce disruptions were categorised and summarised narratively. Thematic analysis of the solutions by three independent reviewers identified nine common themes. Each theme was evaluated using an adapted Ng and de Colombani framework [26] for assessing best practices in public health. Each theme was scored independently by five reviewers (Supplementary Table 2), with a maximum score of 15 representing best practice. The number of countries already implementing relevant measures was recorded by theme.

Data cleaning and analysis for the impact on immunisation were conducted in R (version 3.6.3) and reasons for disruption and solutions were analysed in Excel (Microsoft Corporation, Office 365 Pro Plus). Results are reported as median (interquartile range [IQR]) unless otherwise specified.

### 2.4 Role of the funding source

This study was funded by Sanofi Pasteur. Employees of the sponsor designed the study; were involved in the collection, analysis, and interpretation of data; wrote the outline and reviewed drafts; and were involved in the decision to submit the paper for publication.

## 3 Results

Countries reported on the impact of COVID-19 disruptions for between 13 and 19 antigens, across up to five age groups, as relevant per antigen. Antigens were routinely delivered in 2-19 countries, with ten of the antigens routinely delivered in all 19 participating countries. The antigens reported as routinely delivered by each country are listed in Supplementary Table 3. Eighteen (95%) of the 19 participating countries reported disruption to routine vaccination with at least one antigen. In total, when antigens were counted individually for each age group and each country, 665 were reported as disrupted across the 19 countries and five age groups.

### 3.1 Percentage and number of antigens impacted by country

**Fig. 1:**
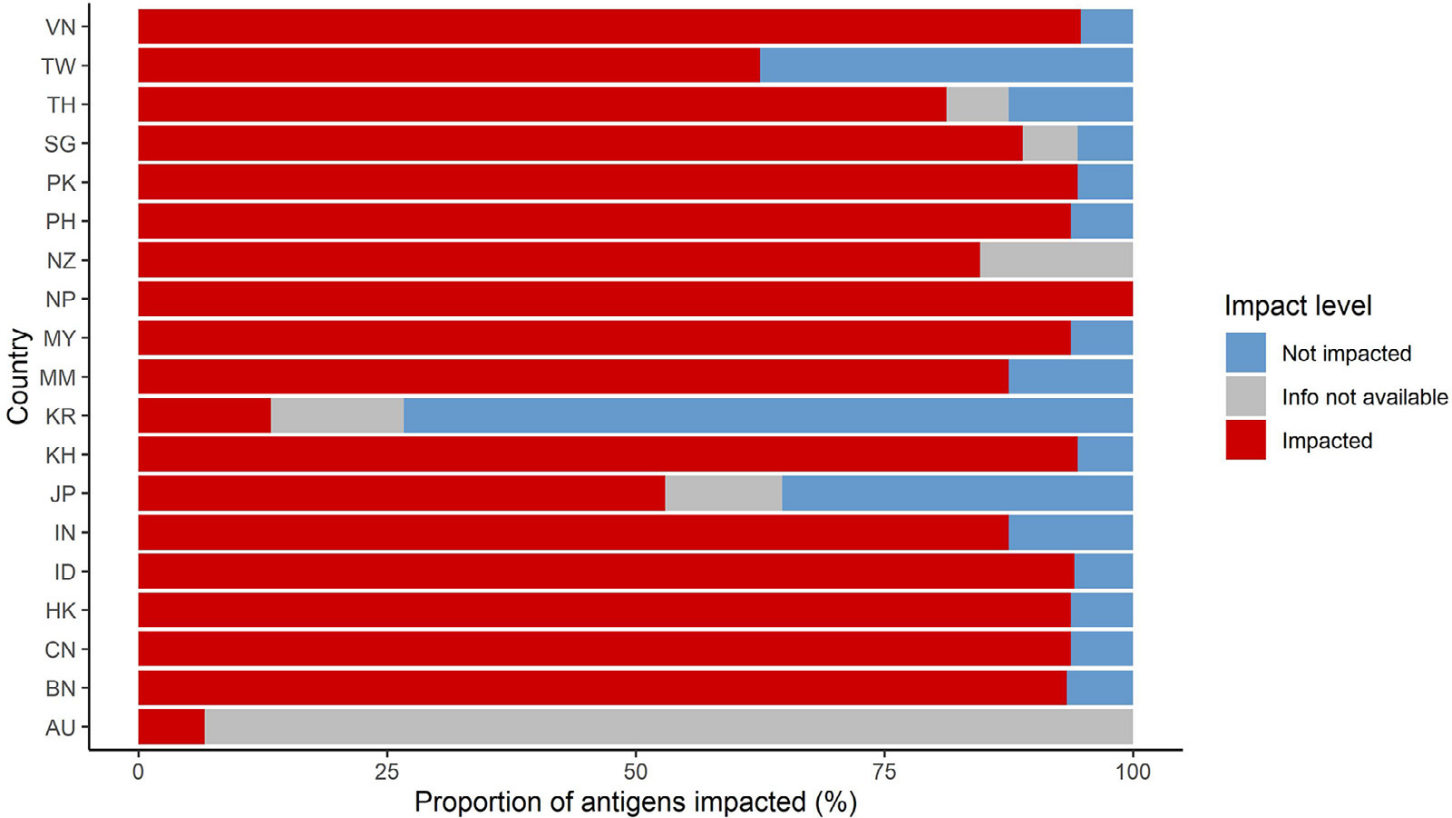
Proportion of antigens impacted by country. The denominator was defined as number of antigens for whom countries answered anything except ‘not applicable’. Median and IQR values overall are presented in the text (Section 3.1). AU, Australia; BN, Brunei; CN, China; HK, Hong Kong; ID, Indonesia; IN, India; JP, Japan; KH, Cambodia; KR, South Korea; MM, Myanmar; MY, Malaysia; NP, Nepal; NZ, New Zealand; PH, Philippines; PK, Pakistan; SG, Singapore; TH, Thailand; TW, Taiwan; VN, Vietnam.

A median of 91% (IQR 77–94%, range 7–100%) of antigens were impacted by country (Fig. 1), which is equivalent to a median of 15 antigens (IQR 11–16) disrupted per country (Supplementary Figure 2). Except for Australia and South Korea, all countries had at least half of their recommended antigens impacted.

**Fig. 2:**
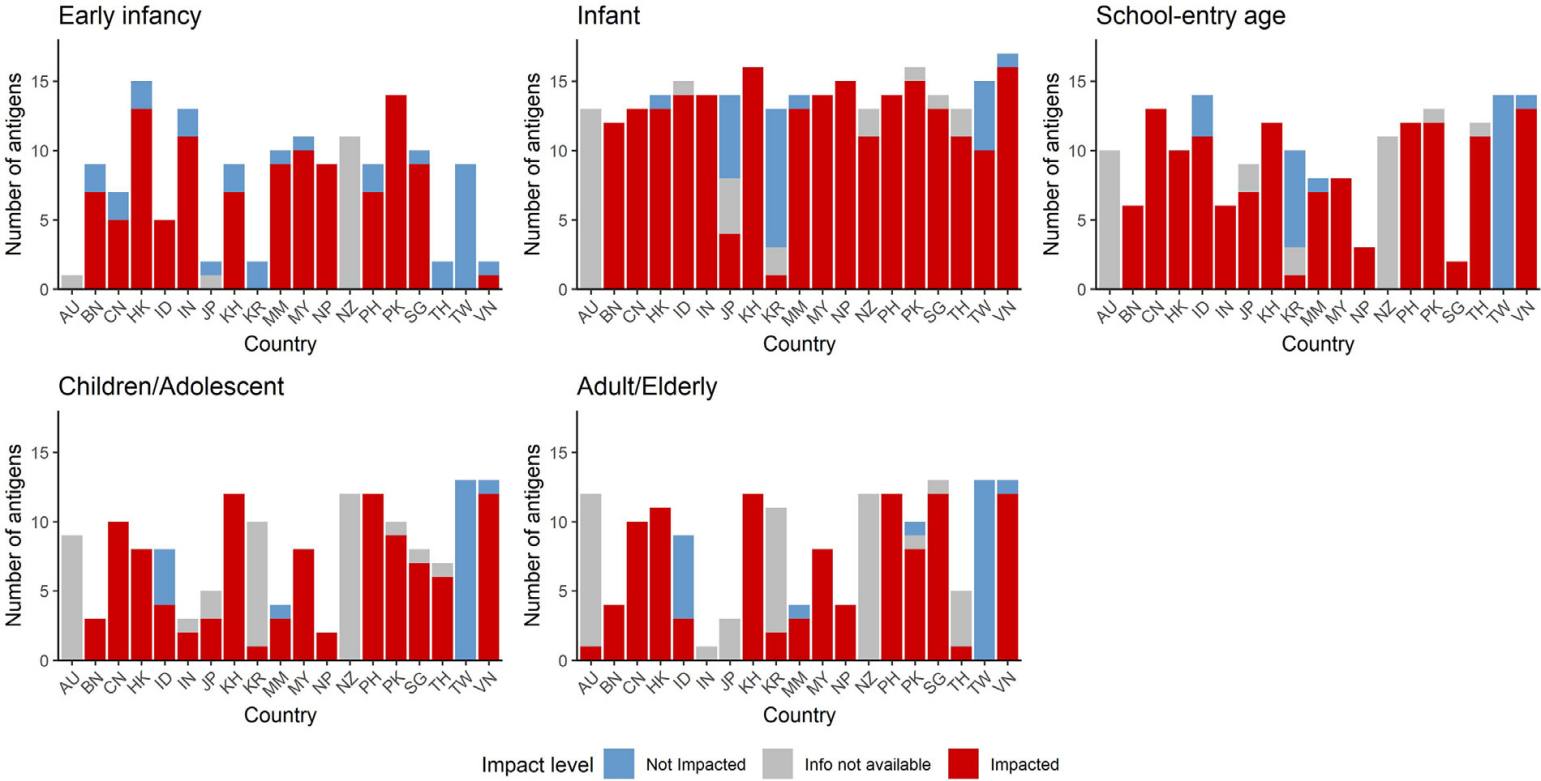
Number of antigens impacted by country and by age Median and IQR values overall are presented in the text (Section 3.1). AU, Australia; BN, Brunei; CN, China; HK, Hong Kong; ID, Indonesia; IN, India; JP, Japan; KH, Cambodia; KR, South Korea; MM, Myanmar; MY, Malaysia; NP, Nepal; NZ, New Zealand; PH, Philippines; PK, Pakistan; SG, Singapore; TH, Thailand; TW, Taiwan; VN, Vietnam.

When stratified by both age and country, disruptions to antigens occurred as follows, shown as median (IQR): early infancy 78% (0–90%); infancy 93% (80–100%); school-entry age 93% (61–100%); children and adolescents 87% (40–100%); and adults/elderly 78% (6–100%). Noting that a greater number of antigens are typically delivered in early infancy and infancy compared with other age groups, this was equivalent to six (0–9), 13 (11–14), seven (2–11), four (2–8), and four (1–10) antigens impacted in each age group, respectively (Fig. 2).

A significant difference was observed between age groups for both number and percentage of antigens impacted (K–W *p*-value <0•05), with subsequent Dunn with Bonferroni correction significant (adjusted *p*-value <0•05) for infancy versus adult/elderly vaccination.

The proportion of antigens impacted by WB income level was 63% (13–89%) in HI countries, 94% (81–94%) in UMI countries, and 94% (88–95%) in LMI countries, equivalent to ten (2–14), 15 (13–15) and 16 (14–17) antigens impacted, respectively. A significant difference was observed by country income level (K–W *p* < 0•05), with Dunn test significant for HI versus LMI countries.

### 3.2 Countries with disruptions by antigen

**Fig. 3:**
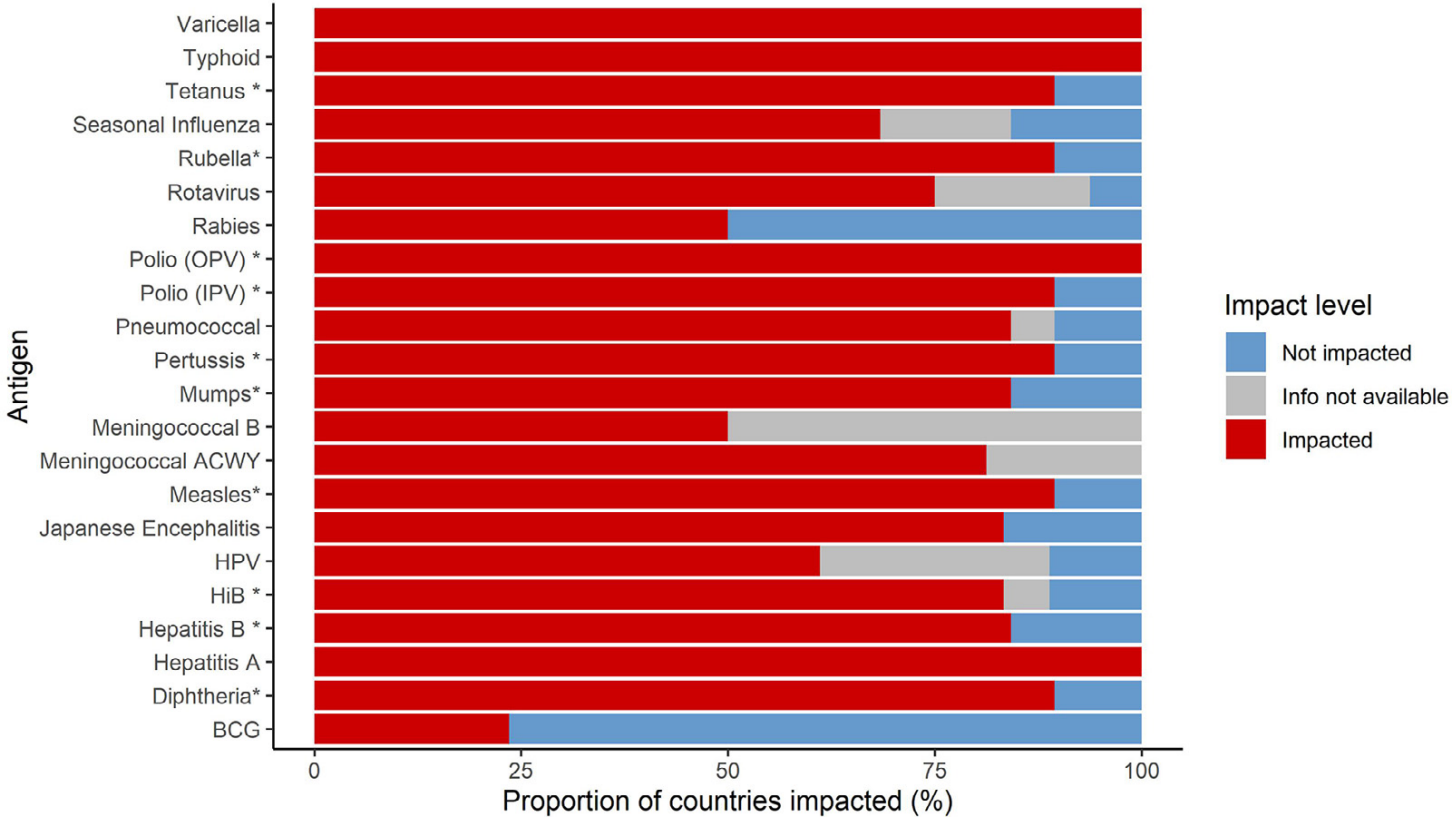
Proportion of countries impacted by antigen Median and IQR values overall are presented in the text (Section 3.2). BCG, bacillus Calmette-Guérin; HiB, *Haemophilus influenzae* B; HPV, human papilloma virus; IPV, inactivated polio vaccine; OPV, oral polio vaccine. *denotes antigens potentially within combination vaccines.

The proportion of countries experiencing disruption is shown by antigen in Fig. 3. With this stratification by antigen, for any given antigen a median of 84% (IQR 72–89%) of countries were impacted, equivalent to a median of 13 (IQR 4–17) countries. This varied by country income level: 46% (37–50%) in HI countries, 88% (66–100%) in UMI countries, and 100% (IQR 89–100%) in LMI countries, with a significant difference between income levels (K–W <0•05, all Dunn tests <0•05). Vaccinations with all antigens except bacillus Calmette-Guérin (BCG) were disrupted in at least half of the countries routinely delivering the relevant vaccines.

Diphtheria, tetanus, pertussis, measles, and rubella vaccines, as well as inactivated polio vaccine (IPV), were impacted in 17 countries. HepB (birth and primary), mumps, and pneumococcal vaccines were impacted in 16 countries, and meningococcal A (either the single monovalent Men A or the MenACWY vaccine) in 13 countries. Oral polio vaccine (OPV), hepatitis A, typhoid, and varicella vaccines were impacted in all countries that routinely deliver them, which is 12 countries for OPV and three to four countries for the others. BCG was the least affected antigen, with disruption reported by only four out of 17 countries that routinely deliver BCG. The next two least affected antigens were rabies and meningococcal B. The total population size of impacted countries is shown by antigen in Supplementary Fig. 3.

When stratified by age groups, the median number of countries affected per antigen was seven for early infancy, 11 for infancy, eight for school-entry-age, four for child/adolescent and three for adult/elderly vaccination. There was a significant difference by age (K–W <0•05), with the number of countries disrupted significantly different in the infant age group compared with each of the other age groups (Dunn-adjusted *p* < 0•05).

### 3.3 Public and private healthcare settings, and geographical heterogeneity

Among all the disrupted antigens counted individually by age group and country and with data available (*N* = 597), 79% were disrupted in the public sector, 83% in the private sector, and 63% in both sectors (Supplementary Fig. 4).

Where both public and private sectors were reported as impacted (*N* = 374), 87% of the antigens impacted were estimated as majority (i.e. >50%) public sector delivery/market share. Where only the private sector was reported as impacted (*N* = 123), 91% of the antigens impacted were estimated as majority private sector delivery/market share. Where only the public sector was reported as impacted (*N* = 100), all of the antigens impacted were estimated as majority public sector delivery/market share.

Seven countries reported within-country geographical differences in disruptions. Socio-economic and geographical disparities accounted for the differences in four countries (e.g., differences were seen in Maori populations in New Zealand, minority and low-income populations in Vietnam, and rural populations in Myanmar, Vietnam, and Cambodia). Heterogeneity in COVID-19 burden and measures, such as regional lockdowns or high transmission areas, accounted for the differences in China, India, and Thailand.

### 3.4 Vaccination coverage rate

Changes in VCR were reported in eight countries; four (Australia, Korea, Thailand, Taiwan) [27], [28], [29], [30] used publicly reported data and four (China, India, Hong Kong, Myanmar) reported estimates based on sales data, clinician interactions, or press releases. The median absolute VCR reduction was estimated as 6% (IQR 3–7%) based upon reported data alone, and 18% (6–79%) when estimates were included.

The absolute reduction in VCRs for four antigens is shown in Table 1. DTP and OPV in infancy were particularly affected, though data were only available for a limited number of countries.

**Table 1:** Reduction in vaccine coverage rates (VCR) for selected antigens representing highly outbreak-prone diseases.

Antigen	Absolute percentage reduction in VCR: median % (IQR)
DTP, Infancy, *n* = 6	42^a^ (12–79)
OPV, Infancy, *n* = 3	79 (42–79)
School-entry age, *n* = 1	4
IPV, School-entry age, *n* = 2	29 (4–53)
Measles, School-entry age, *n* = 4	9 (3–31)

a 70% when weighted by birth cohort size. DTP, diphtheria, tetanus and pertussis; IPV, inactivated polio vaccine; IQR, interquartile range; n, number of countries responding; OPV, oral polio vaccine.

Australia reported a 17% absolute increase in VCR for influenza (adults/elderly), and South Korea reported a 1% increase in VCR for HepB and BCG (early infancy), and measles and pneumococcus (infancy).

### 3.5 Return to pre-COVID-19 level

Of all antigens by age group and country reported as impacted, only 29% had returned to pre-COVID-19 coverage by 1 June 2020; 39% remained impacted, and for 32% the situation was unknown. DTP, HepB, MenACWY, pneumococcal and polio (both OPV and IPV) vaccinations were reported as the highest number not yet recovered on 1 June 2020.

### 3.6 Reasons for disruption

**Fig. 4:**
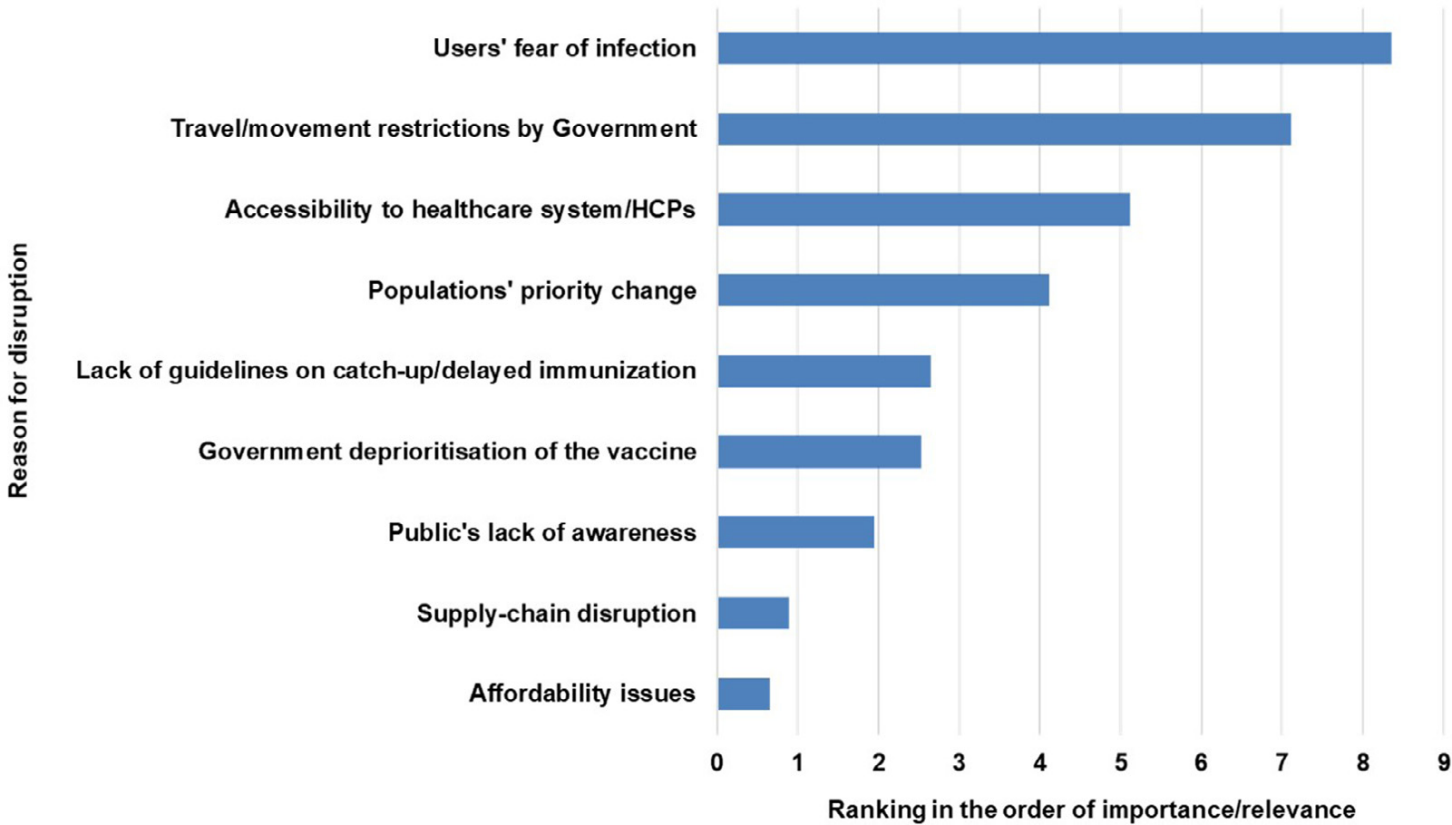
Weighted average rank of reasons for disruption Score of nine is highest-ranked reason for disruption, zero represents not stated as a reason. HCP, healthcare provider.

The highest-ranked reasons for disruption were fear of infection, movement/travel restrictions, and limited healthcare access (Fig. 4). These were also the most frequently cited reasons, reported by 100%, 88%, and 76% of countries, respectively. Supply-chain disruption and affordability issues were the two lowest-ranked causes.

### 3.7 Solutions

Nine major themes were identified, initiated by four sectors (government, medical societies, HCPs, and/or industry) and targeting two main stakeholders (HCPs and the general public) (Table 2).

**Table 2:** Solutions reported by the countries, including the best practice score as assessed using a modified Ng and de Colombani's framework [26], number of countries providing examples of real-world implementation, and implicated providers and stakeholders.

#	Themes	Best practice score^a^	Number of countries reporting examples of use	Sectors which are implementing the solution	Target stakeholders for solution
1	Separating the healthy group from the rest at the time of immunisation	13	3	HCPs	HCPs, Public
2	Non-traditional or separate vaccination venues	12	5	HCPs	HCPs, Public
3	Virtual engagement with stakeholders	12	17	All	HCPs, Public
4	Social media campaigns to general public	12	4	All	Public
5	Guidelines on immunisation	11	9	Govt	HCPs, Public
6	Immunisation recommendations published	8	5	Med societies	HCPs
7	Categorising immunisation as essential vs non-essential medical service	7	1	Govt	HCPs
8	Securing supply chain by industry	6	1	Industry	HCPs
9	Government's transparency on communicating immunisation/VCR details	5	2	Govt	HCPs, Public

a Assessed using a modified Ng and de Colombani's framework; see Supplementary Table 2 Govt, government; HCPs, healthcare providers; med, medical.

The best practice scores for each theme ranged from 5/15 to 13/15. Examples of real-world implementation of practices within themes were reported in between 1/19 (rarest) to 17/19 (widely used) countries. The highest best practice scoring theme was separation of healthy vaccination groups from unwell patients, followed by non-traditional vaccination venues, social media campaigns and virtual engagements (Table 2). However, only one high-scoring solution – virtual engagement – was reported to be commonly used by the participating countries (Table 2).

Examples of applications of some of the solutions are shown in Table 3. Several countries leveraged social media and mass media to communicate to otherwise difficult-to-reach patients. India, Singapore, and Thailand described how they had separated healthy groups from the rest at the time of immunisation, generally by providing dedicated spaces or times. Several countries reported the use of non-traditional or separate vaccination venues, such as drive-through or home vaccination, offered primarily by the private sector.

**Table 3:** Examples of solutions implemented to minimise disruption of immunisation.

Country	Action taken
**Virtual engagement with stakeholders by industry, government, and/or medical societies**	
Many	• Virtual communications (webinars, conferences, meetings, emails) to HCPs from industry
Many	• Awareness campaigns on the importance of immunisation, immunisation guidelines during the pandemic, and the effects of delayed vaccination via digital and mass media channels (TV, radio, webinars, TV advertisements, etc) by governments to the public and HCPs
**Separating the healthy group from the rest at the time of immunisation**	
India	• Hospitals created a dedicated immunisation space away from unwell patients
Singapore	• Private health clinics and hospitals dedicated clinic hours/sessions solely for immunisation• In private health clinics and hospitals some paediatricians delayed childhood vaccinations within the permissible time interval to ensure parents would be comfortable in bringing their children to receive their subsequent shots
Thailand	• Well baby and well adult clinics were created in hospitals• An appointment system was initiated to separate the sick from the well• Requirement to call for appointment before vaccination
**Use of non-traditional or separate vaccination venues**	
Hong Kong	• Some clinics offered a home vaccination service, and suggested patients group together for this in order to lower the cost
Malaysia	• Private GPs and paediatricians implemented drive-through vaccinations at their practices• Some also implemented home vaccination
Myanmar	• The private sector started home vaccination services
Philippines	• Government healthcare workers went house-to-house for vaccination in areas permitted or not under lockdown• The private sector offered drive-through and home vaccination
Thailand	• Private hospitals provided drive-through vaccination• Immunisation services were provided at home• Vaccine Safe Zone project was initiated by Sanofi Pasteur after Thai government declared State of Emergency

GP, general practitioner; HCP, healthcare provider.

## 4 Discussion

In this study, 95% of participating SEAR/WPR countries reported vaccination disruption. A median of 91% of antigens were impacted by country. Infant and school-age vaccinations were most affected, and both public and private sector healthcare providers experienced disruptions. Coverage had not recovered for 39% of impacted antigens by 1 June 2020. Fear of infection, movement/travel restrictions and limited healthcare access were the highest-ranked reasons for disruption. Assessment of solutions identified several solutions scoring highly for best practice but reported to be used by only a few countries, which could potentially be adapted and deployed elsewhere.

Our findings suggest that disruptions to routine vaccination are more widespread in SEAR/WPR (95% of 19 participating countries) than the 45–55% reported for the region in 11 countries surveyed by WHO in June 2020 [9]. A third WHO survey up to July 2020 asked respondents from 91 countries in five WHO regions to report disruptions to immunisation services as none, partial or severe. Disruptions were reported to outreach immunisation services in 70% of countries (severe disruption in 18%) and facility-based services in 61% (severe disruption in 10%) [10]. These data included but were not limited to SEAR/WPR.

The first WHO pulse survey reported on the number of countries in SEAR/WPR affected for routine immunisation by antigen as follows: eight countries with measles-mumps-rubella (MMR) disruption, seven OPV, one IPV and tetanus, and no MenA or typhoid disruptions at 29 April 2020, with MMR disruptions revised to four countries in the June 2020 pulse survey [8,9]. In contrast, our results indicated that MMR was disrupted in 16 countries in the SEAR/WPR region, IPV and tetanus in 17, OPV in 12, MenA (either single or ACWY vaccine) in 13, and typhoid in three. Overall, we found that a median of 84% of countries were impacted for any given antigen. This is the first report to assess disruption of commonly used antigens in SEAR/WPR. We found that most participating countries reported disruption in most antigens, highlighting the need for action.

Disruption was highest in the infant and school-entry age groups, both in terms of numbers and proportion of antigens impacted. Given the large number of vaccines recommended in early infancy, substantial disruption may also have been expected in this group; this was not observed, likely due to many births taking place in health facilities, thus maintaining access to vaccination. These results suggest that subsequent follow-up visits for infant and childhood vaccination pose a challenge, leading to missed doses. Solutions that can improve coverage in these two age groups are needed.

This is to our knowledge the first study to explore vaccination disruption by public and private healthcare sector provision. In both sectors, a similar percentage of antigens were disrupted. We cannot convert these percentages into absolute numbers without knowing how many vaccines each sector delivers, but it is clear that both sectors were substantially disrupted. This is important because efforts that focus only on restoring vaccines delivered by the public sector will miss a key source of disruption. Government and private healthcare providers should work together towards restoring VCR back to pre-pandemic levels as rapidly as possible. Indeed, it had already been suggested prior to COVID-19 that cooperation between private providers and national immunisation programmes should be increased [31].

More antigens were disrupted in middle-income than in high-income countries. Seven countries also reported within-country geographical differences in disruption; two of these were countries with large populations (China and India). A recently published study from Pakistan also demonstrated greater disruption in rural than urban population [19]. These results highlight the risks of missing important variations when summarising disruptions at a regional level, and demonstrate the need for VCR data and appropriate tailored solutions at the national or even sub-national level. The variations in the impact of COVID-19 by age, national income category, and within countries revealed by our study provide pointers for targeted action.

From the VCR differences reported by eight countries, we estimated a median 6% absolute reduction based upon publicly reported data and 18% once estimates were included. Reported data tend to be available from higher-income countries with more robust public healthcare systems, and are thus likely to be the least impacted in the region. The higher estimates, although limited by data quality, may be more reflective of the reality in SEAR/WPR.

The estimated VCR reduction for DTP in our study (42%) was similar to that estimated by WHO for the region (approx. 40–50% relative reduction 2019–2020 in SEAR/WPR) and GAVI in Pakistan (49%) [18,32]. There were large reported reductions in OPV and IPV uptake, which could negatively impact the global polio eradication initiative. However, these results should be interpreted with caution given the uncertain quality and strong influence of several large countries.

Disruptions in vaccine coverage are likely ongoing, as 39% of disrupted antigens had not returned to pre-COVID levels by 1 June 2020, and the situation was unknown for 32%. Data availability and quality for VCRs remains limited. More robust and real-time reporting of VCRs is needed, not just for this pandemic, but also to potentially improve vaccine coverage beyond COVID-19.

The most cited reasons for disruption were fear of infection, movement/travel restrictions, and limited accessibility to healthcare. These are in line with reasons reported to WHO in June 2020 [9]. Among those respondents, 48% cited users’ concern about the risk of exposure to COVID-19, and 33% cited transport/lockdown issues, as reasons for disruption. A snapshot survey conducted by IMPRINT in April 2020 found that more than 50% of countries reported disruptions to either maternal or infant/toddler vaccine delivery, although only two SEAR/WPR countries were included [11]. The reasons given were grouped into three themes: access issues (including travel restrictions), provider issues (including staff shortages and vaccine supply problems), and user concerns (primarily fear of infection and broader vaccine hesitance) [11].

In the current survey, reasons for disruption did not differ greatly between country income levels, suggesting that solutions to tackle these disruptions could potentially be adapted for use between countries.

We used a reference framework to systematically assign a best practice score to nine overall themes covering proposed solutions. The highest-scoring initiatives included separating healthy groups from other patients or using separate vaccination venues – initiatives that may be easily deployable to other countries and address fear of infection, which was the top-cited reason for disruption. However, few countries reported implementing these actions.

The most widely used solution was the use of virtual engagement of patients and HCPs. Another solution, social media campaigns, is not a new concept for supporting vaccination campaigns, but was not reported in the survey as widely used despite high smartphone ownership in the region and the speed and low cost involved. Social media and TV campaigns are vital to ensure that users understand the importance of not missing scheduled vaccinations and are aware of new measures to ensure safe access to vaccination. Furthermore, the role of the traditional media should not be overlooked.

The IMPRINT survey of maternal, neonatal, and infant vaccination [11] also identified proposed solutions to overcome disruptions, in some cases similar to those proposed in our study. The proposed solutions included targeted communication strategies to reinforce the importance of routine immunisations during the pandemic. The need for context-specific, rapidly adaptable methods of delivering vaccines was also mentioned. The authors called for further quantification of routine vaccination disruption; our results represent a response to this identified evidence gap in the literature.

Our study has many strengths, including its wide coverage of SEAR/WPR countries, representing 94% of the population of the region; the use of multiple information sources and information gathering by teams of known respondents to reduce the risk of single-responder bias; and the inclusion of data on vaccine provision by the private healthcare sector and within-country heterogeneity. Further strengths are the exploration of solutions and the use of a framework to identify best practices among these solutions.

Limitations of this study include its reliance on self-reporting – a limitation shared by other surveys on this topic. Data were not sought from the service providers themselves, and only countries with a Sanofi Pasteur medical team were included; however, regional coverage remained high. To minimise potential bias, publicly available data sources were used when possible; academic partners were involved as co-authors; and all relevant antigens were included, not just Sanofi Pasteur products. In addition, the degree to which services were impacted was not quantified, except in part for vaccination coverage rates, where data were sparse. Further limitations relate to the limited quality, availability, and reliability of some data, and the timing of the survey relatively early in the pandemic, which has continued to evolve. Many solutions were still in the implementation phase, so impact of measures was not yet available. Since our survey, SEAR/WPR countries may have implemented measures to rectify vaccine disruptions, and there are anecdotal reports of improvements in vaccination services in some countries in the region in late 2020.

Bearing these shortcomings in mind, we believe that the information nevertheless provides important insights and solutions useful in SEAR/WPR countries and beyond.

## 5 Conclusion

Disruption of routine vaccinations due to COVID-19 is more widespread in SEAR/WPR countries than previously reported, and has impacted both public and private sector vaccination. Key reasons cited were users’ fear of becoming infected if they attended vaccination visits, movement/travel restrictions, and limited healthcare access. Several solutions scoring highly for best practice were reported to be used by only a few countries, so there may be value in exploring their potential for use elsewhere. Future waves of the pandemic could cause ongoing and increased disruption to vaccination services. Governments and private providers need to act urgently to implement solutions, to avoid the possible resurgence of vaccine-preventable diseases.

## Contributors

RCH, GN, YC, PC, MCN, and AC designed this study; KVK and KM were involved in questionnaire piloting; RCH, GN, YC, PC, and AA analysed the data; AS, AOL, and CPC provided interpretation from a country perspective; all authors were involved in interpretation of the study results, review and revision of drafts, and approval of the article prior to submission.

## Data sharing statement

The relevant data from this survey are contained in the article and supplementary material.

Editor note: The Lancet Group takes a neutral position with respect to territorial claims in published maps and institutional affiliations.

## Declaration of Competing Interest

At the time of the preparation of this work, Rebecca C Harris, Yutao Chen, Pierre Côte, Antoine Ardillon, Maria Carmen Nievera, Kiruthika Velan Kandasamy, Kuharaj Mahenthiran, Ta-Wen Yu, Changshu Huang, Clotilde El Guerche-Séblain, Juan C Vargas-Zambrano, Ayman Chit, and Gopinath Nageshwaran were employees of Sanofi Pasteur and might be shareholders of Sanofi. Anna Ong-Lim and Somasundaram Aiyamperumal have no disclosures. Poh Chong Chan reports speaker fees and other fees for involvement in clinical trial from Sanofi Pasteur.

## Editor note

The Lancet Group takes a neutral position with respect to territorial claims in published maps and institutional affiliations.

## Appendix Supplementary materials

Image, application 2
