# Supplementary material for: Impact of COVID-19 on routine immunisation in South-East Asia and Western Pacific: Disruptions and solutions
Source: Lancet Reg Health West Pac. 2021 Apr 21;10:100140. doi: 10.1016/j.lanwpc.2021.100140 (PMC8057868; doi:10.1016/j.lanwpc.2021.100140)
Supplement: Supplementary file 1 [file mmc1.pdf]

Impact of COVID-19 on routine immunisation in South-East Asia and Western Pacific: disruptions and solutions

Online appendix

Supplementary Figure 1: Countries that participated in the survey are shown in green. Map created with Mapchart.net.

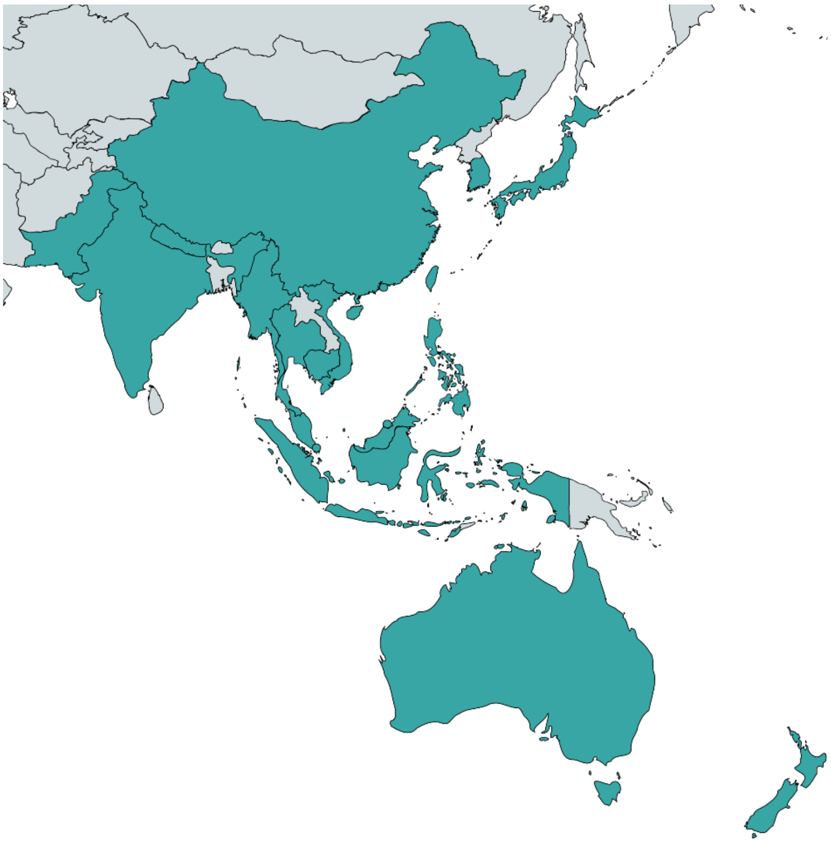

Supplementary Figure 2: Number of vaccines impacted in each country

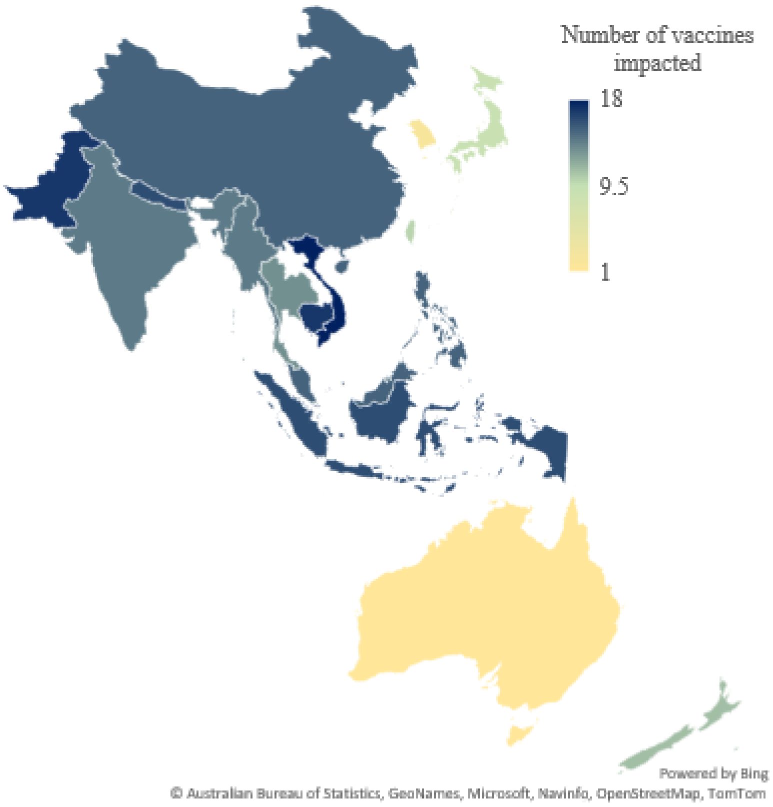

Note that South Korea appears cut off from mainland Asia, as North Korea was not included in the survey. Countries not participating in the survey are not included in the map.

Supplementary Figure 3: Total all-age population (millions) in countries reporting each vaccine impacted.

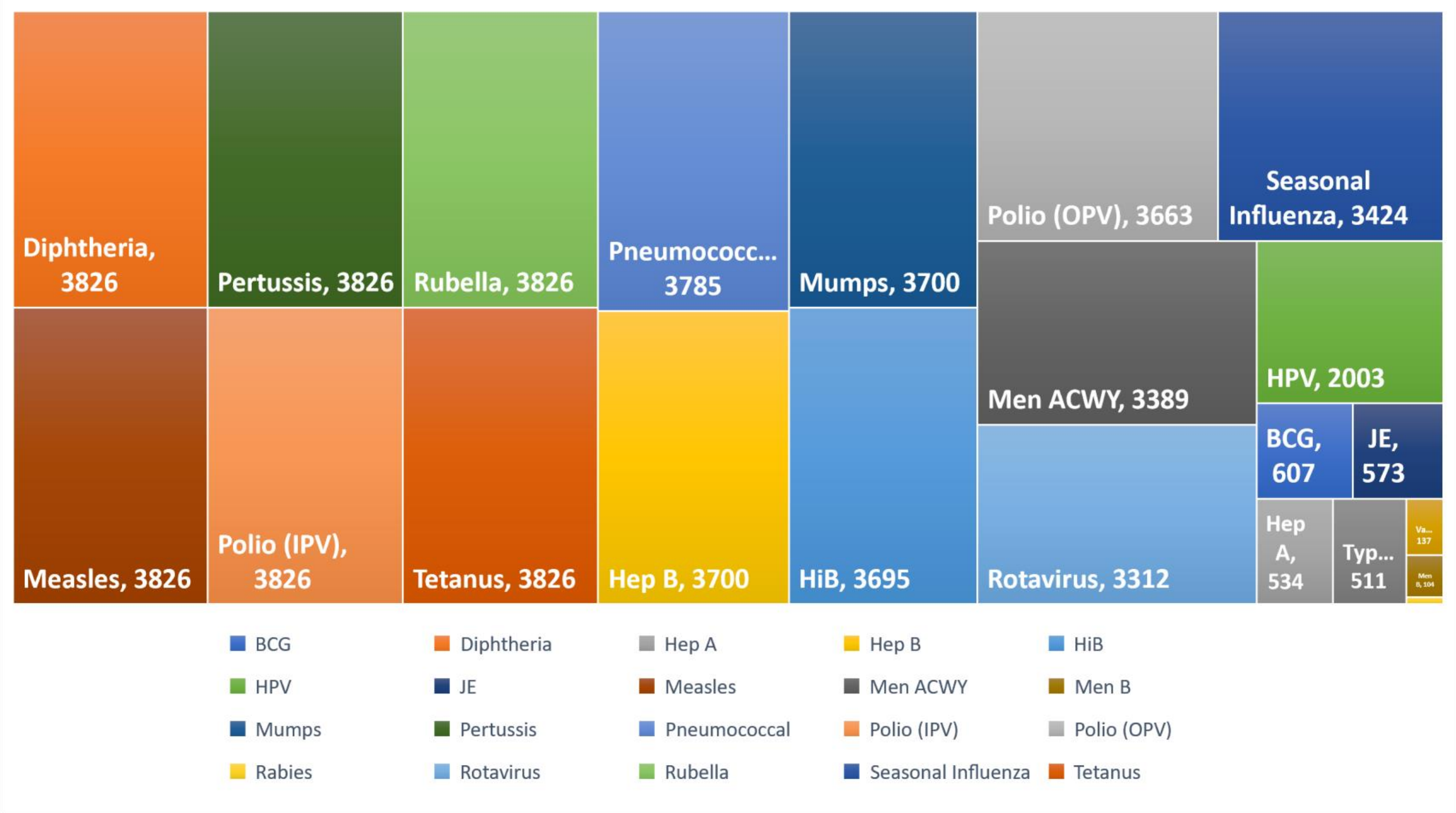

It should be noted that the represented populations do not account for ages targeted for routine vaccination, nor do they account for the age groups reported as impacted. The intention is to provide an indication of the relative scale of the population potentially at risk if declines in vaccination coverage rates were to lead to increases in transmission of vaccine preventable diseases.

Supplementary Figure 4: Number of antigens impacted in the public and private healthcare settings. Antigens were counted individually for each age group and each country

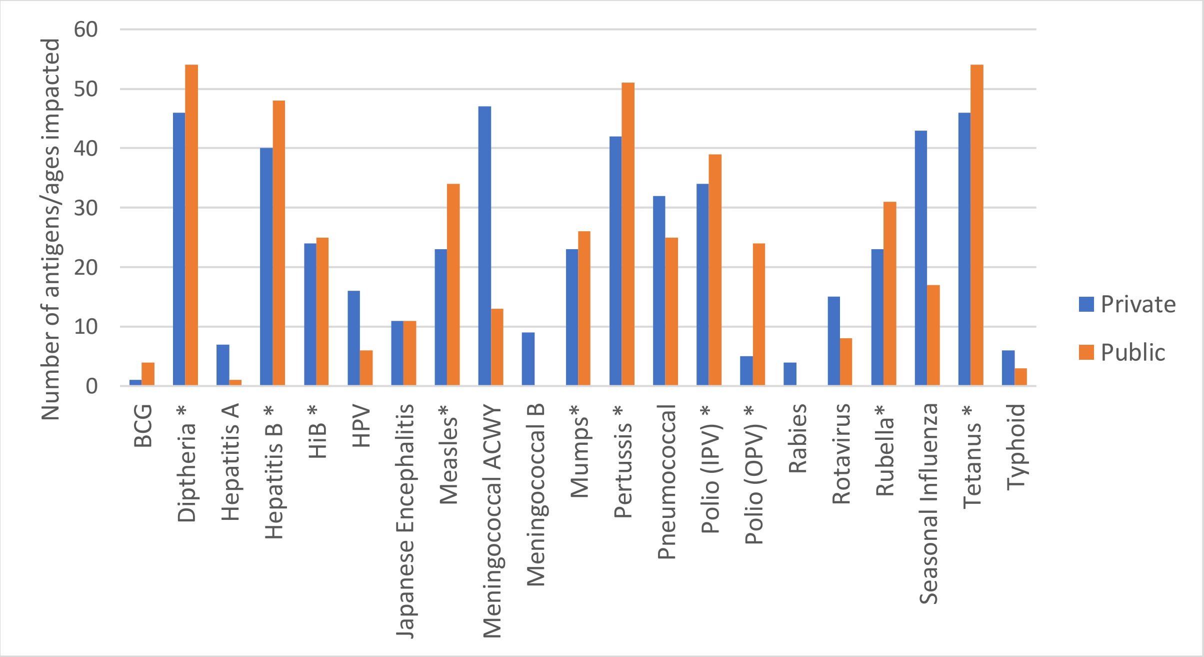

BCG, bacillus Calmette-Guérin; HiB, *Haemophilus influenzae* B; HPV, human papilloma virus; IPV, inactivated polio vaccine; OPV, oral polio vaccine

Supplementary Table 1: Impact of COVID-19 on Routine Immunisation Practices questionnaire as circulated to countries

**Section 1: Country Information**
**Country (please fill out one form per country)**
**Population size** (2019, in millions, e.g. 2.43)
**Annual birth cohort** (2019, full number, e.g. 340,200)

Early infancy (0–8 weeks), infants and toddlers (9 weeks to 23 months; subsequently referred to as ‘infancy’), preschool and school-entry age (2–6 years; subsequently referred to as ‘school-entry age’), children and adolescents (7–17 years), and adults and elderly

**Section 2: Vaccines with COVID-related disruption**									
*** If delivered as part of a combination vaccine, please fill all relevant rows for the antigens in that vaccine**	**Have the following immunization practices been impacted by COVID disruptions in your country?** By ‘impacted’ we mean a change in the number of people receiving the vaccine during COVID-19 even relatively small disruptions are relevant, the scale of disruption will be captured in the next section. Please fill in all boxes; if not used in a given age group in your country, please select ‘not applicable’.					**Is this vaccine usually recommended or optional according to government policy?**	**Was this vaccine prioritised according to government policy or medical society recommend-dations during COVID?**	**What proportion of the public/ private market split for this vaccine is <u>public</u>? (public market %, please provide single number <u>not</u> range)**	**Has the public or private or both markets been impacted for this vaccine?**
	**Early infancy (0 to 8weeks)**	**Infants and toddlers (9w to <24m)**	**Pre-school and school-entry age (2y to <7y)**	**Children and adolescents (7 to <18y)**	**Adults and elderly (18y+)**				
BCG									
Diphtheria *									
Tetanus *									
Pertussis *									
Hepatitis B *									
Polio (IPV) *									
Polio (OPV) *									
HiB *									
Pneumococcal									
Rotavirus									
Measles*									
Mumps*									

Rubella*									
HPV									
Meningococcal B									
Meningococcal ACWY									
Seasonal influenza									
Other (please add):									
Other (please add):									

Please indicate which DTP-combination vaccines are in use in your country (delete those not in use):	Hexavalent (DTaP-HepB-Hib-IPV) Tdap<br>Hexavalent (DTwP-HepB-Hib-IPV) Tdap-IPV<br>Pentavalent (DTwP-HepB-Hib) DT<br>Pentavalent (DTaP-IPV-Hib) Td
[Optional]: Further details are not essential, but please share additional information here *only if* there is information about your programme that are *needed to understand the data shared above* (e.g. differences in vaccines used for the same disease in public vs private sector, an increase in uptake during COVID, seasonality of vaccination programmes):	

**Section 3: Vaccine Coverage Rate (VCR) Details**							
	**What was the BASELINE VCR for each vaccine before COVID-19 disruptions?** (in % terms, whole numbers, NO RANGES please), enter 'INA', if info not available					**Please indicate whether this is estimated or reported**	**<u>If reported</u>, please paste links/references to any reported data.**<br>**<u>If estimated</u>, please indicate (where possible) if any information this was based on (e.g. sales data, clinician interactions, etc)**
Cell will grey-out if 'not applicable' or 'info not available' answered to Section 2	**Early infancy (0 to 8weeks)**	**Infants and toddlers (9w to <24m)**	**Pre-school and school-entry age (2y to <7y)**	**Children and adolescents (7 to <18y)**	**Adults and elderly (18y+)**		
	Same list of antigens as in Section 2						

	**What was the VCR during the PEAK of COVID-related disruptions?** (in % terms, whole numbers, NO RANGES please), enter ‘INA’ if info not available.					**Please indicate whether this is estimated or reported**	**If reported, please paste links/ references to data. If estimated, please indicate (where possible) any information this was based on**	**Have the public/ private markets been similarly affected?**	**Please briefly describe any differences in disruptions in public vs private market**	**Were there any geographical/ regional differences in the impact on VCR?**	**If yes, please briefly describe how they differed (e.g. urban more impacted than rural, a particular province more impacted)**	**Has the VCR returned to normal as of 1st June 2020?**
	**Early infancy (0 to 8weeks)**	**Infants and toddlers (9w to <24m)**	**Pre-school and school-entry age (2y to <7y)**	**Children and adolescents (7 to <18y)**	**Adults and elderly (18y+)**							
	Same list of antigens as in Section 2											

**Section 4: Reasons for Disruption**		
	**Please indicate which of the below were a reason for disruption of the coverage of routine immunisation?**	**Please rank these reasons in order of importance/relevance**
**Supply-chain disruption**		
**Travel/movement restrictions by Government**		
**Consumers' fear of infection**		
**Consumers' priority change**		
**Accessibility to healthcare system/HCPs**		
**Affordability issues**		
**Consumers' lack of awareness**		
**Government deprioritisation of the vaccine**		
**Lack of guidelines on catch-up/delayed immunization**		
**Others, please list:**		

Section 5: Measures to reduce vaccination disruption			
**Please share examples of measures undertaken by different stakeholders to minimise vaccination disruption**	**Has this measure been implemented already?**	**Has the impact of this measure been evaluated?**	**Where impact has been evaluated, please share the summarised results of the evaluation.**
Please share examples of public health measures undertaken by the Government or public health bodies to minimize vaccination disruption (e.g. policy, programmes). Please focus on examples that were successful, and provide a summary of the measure taken, and indicate any key characteristics of the measure that were important to its success.			
Please share examples of private health sector initiatives to minimize vaccination disruption (e.g. patient journey innovation such as vaccination drive throughs in Philippines). Please focus on examples that were successful, and provide a summary of the measure taken, and indicate any key characteristics of the measure that were important to its success.			
Please share examples of measures undertaken by the Sanofi Pasteur team (or other vaccine companies) to minimize vaccination disruption (e.g. Bilateral ZOOM meeting between CH & PH; Medical 360 by DE; Vaccines Confidence Initiative by ES). Please focus on examples that were successful, and provide a summary of the measure taken, and indicate any key characteristics of the measure that were important to its success.			
If you have any examples of measures taken that were unsuccessful in minimising disruption, please provide a short description, and any key characteristics that may have contributed to the lack of success.			

Section 6: Other information of relevance	
Please feel free to share any other information of importance/relevance to the subject of this questionnaire	

Supplementary Table 2: Modified Ng and de Colombani's framework[1] for assessing the fitness to purpose of proposed solutions

Category	Criterion	Question	Yes	No
**Context**	**Relevant**	Is this initiative relevant to the COVID-related situation? (Based on top three reasons for disruption)	1	0
		Is this initiative relevant to the setting of the jurisdictions during COVID-related situation? (income level, geographical barriers, etc)	1	0
**Process**	**Community participation**	Have the members of the community been involved? (Public and private side)	1	0
		Was the synergy with community participation considered during programme development and implementation?	1	0
	**Righteous**	Does it bring tangible benefits outweighing the risk of not doing it?	1	0
		Does it demonstrate respect for individual autonomy and privacy?	1	0
		Have the vulnerable groups been considered?	1	0
	**Replicable**	Is this initiative generalisable to other settings/jurisdictions?	1	0
	**Timeliness**	Can the initiative be implemented within a period of 1 month?	1	0
**Outcomes**	**Effective**	Can the direct impact be measured for this initiative?	1	0
		Did it achieve desirable outcomes?	1	0
	**Efficient**	Did the initiative use locally accessible resources?	1	0
		Does this initiative use currently existing resources?	1	0
	**Sustainable**	Does it have the potential to continue through local ownership after pandemic?	1	0
		Is it financially viable in the long term (run on its own)?	1	0

[1] Ng E, de Colombani P. Framework for Selecting Best Practices in Public Health: A Systematic Literature Review. *J Public Health Res* 2015;4(3):577

Supplementary Table 3: Vaccines in use in each country.

Country	Population (thousands)	Vaccines in routine use
Australia	25 398	Diphtheria, Hepatitis B, HiB, HPV, Measles, Meningococcal ACWY, Meningococcal B, Mumps, Pertussis, Pneumococcal, Polio (IPV), Rotavirus, Rubella, Seasonal Influenza, Tetanus
Brunei	445	BCG, Diphtheria, Hepatitis B, HiB, HPV, Measles, Meningococcal ACWY, Mumps, Pertussis, Pneumococcal, Polio (IPV), Rotavirus, Rubella, Seasonal Influenza, Tetanus
Cambodia	16 716	BCG, Diphtheria, Hepatitis B, HiB, HPV, Measles, Meningococcal ACWY, Mumps, Pertussis, Pneumococcal, Polio (IPV), Polio (OPV), Rotavirus, Rubella, Seasonal Influenza, Tetanus, Japanese Encephalitis, Rabies
China	1 425 200	BCG, Diphtheria, Hepatitis B, HiB, HPV, Measles, Meningococcal ACWY, Mumps, Pertussis, Pneumococcal, Polio (IPV), Polio (OPV), Rotavirus, Rubella, Seasonal Influenza, Tetanus
Hong Kong	7 548	BCG, Diphtheria, Hepatitis B, HiB, HPV, Measles, Meningococcal ACWY, Meningococcal B, Mumps, Pertussis, Pneumococcal, Polio (IPV), Rotavirus, Rubella, Seasonal Influenza, Tetanus
India	1 383 198	BCG, Diphtheria, Hepatitis B, HiB, HPV, Measles, Meningococcal ACWY, Mumps, Pertussis, Pneumococcal, Polio (IPV), Polio (OPV), Rotavirus, Rubella, Seasonal Influenza, Tetanus
Indonesia	272 223	BCG, Diphtheria, Hepatitis B, HiB, HPV, Measles, Mumps, Pertussis, Pneumococcal, Polio (IPV), Polio (OPV), Rubella, Seasonal Influenza, Tetanus, Hepatitis A, Typhoid, Japanese Encephalitis
Japan	126 496	BCG, Diphtheria, Hepatitis B, HiB, HPV, Measles, Meningococcal ACWY, Mumps, Pertussis, Pneumococcal, Polio (IPV), Rotavirus, Rubella, Seasonal Influenza, Tetanus, Japanese Encephalitis, Varicella
Malaysia	32 869	BCG, Diphtheria, Hepatitis B, HiB, HPV, Measles, Meningococcal ACWY, Mumps, Pertussis, Pneumococcal, Polio (IPV), Polio (OPV), Rotavirus, Rubella, Seasonal Influenza, Tetanus
Myanmar	54 808	BCG, Diphtheria, Hepatitis B, HiB, HPV, Measles, Meningococcal ACWY, Mumps, Pertussis, Pneumococcal, Polio (IPV), Polio (OPV), Rotavirus, Rubella, Seasonal Influenza, Tetanus
Nepal	30 260	BCG, Diphtheria, Hepatitis B, HiB, Measles, Meningococcal ACWY, Mumps, Pertussis, Pneumococcal, Polio (IPV), Polio (OPV), Rubella, Seasonal Influenza, Tetanus, Hepatitis A, Typhoid
New Zealand	4 834	Diphtheria, Hepatitis B, HPV, Measles, Mumps, Pertussis, Pneumococcal, Polio (IPV), Rotavirus, Rubella, Seasonal Influenza, Tetanus, Varicella
Pakistan	208 362	BCG, Diphtheria, Hepatitis B, HiB, HPV, Measles, Meningococcal ACWY, Mumps, Pertussis, Pneumococcal, Polio (IPV), Polio (OPV), Rotavirus, Rubella, Seasonal Influenza, Tetanus, Hepatitis A, Typhoid
Philippines	109 703	BCG, Diphtheria, Hepatitis B, HiB, HPV, Measles, Meningococcal ACWY, Mumps, Pertussis, Pneumococcal, Polio (IPV), Polio (OPV), Rotavirus, Rubella, Seasonal Influenza, Tetanus
Singapore	5 935	BCG, Diphtheria, Hepatitis B, HiB, HPV, Measles, Meningococcal ACWY, Meningococcal B, Mumps, Pertussis, Pneumococcal, Polio (IPV), Rotavirus, Rubella, Seasonal Influenza, Tetanus, Varicella
South Korea	51 507	BCG, Diphtheria, Hepatitis B, HiB, HPV, Measles, Meningococcal ACWY, Mumps, Pertussis, Pneumococcal, Polio (IPV), Polio (OPV), Rotavirus, Rubella, Seasonal Influenza, Tetanus
Taiwan	23 818	BCG, Diphtheria, Hepatitis B, HiB, HPV, Measles, Mumps, Pertussis, Pneumococcal, Polio (IPV), Rotavirus, Rubella, Seasonal Influenza, Tetanus, Hepatitis A, Japanese Encephalitis
Thailand	69 411	BCG, Diphtheria, Hepatitis B, HiB, HPV, Measles, Meningococcal ACWY, Mumps, Pertussis, Pneumococcal, Polio (IPV), Polio (OPV), Rubella, Seasonal Influenza, Tetanus, Japanese Encephalitis
Vietnam	98 360	BCG, Diphtheria, Hepatitis B, HiB, HPV, Measles, Meningococcal ACWY, Meningococcal B, Mumps, Pertussis, Pneumococcal, Polio (IPV), Polio (OPV), Rotavirus, Rubella, Seasonal Influenza, Tetanus, Japanese Encephalitis, Rabies
